# Acceptance and use of complementary and alternative medicine among medical specialists: a 15-year systematic review and data synthesis

**DOI:** 10.1186/s13643-021-01882-4

**Published:** 2022-01-14

**Authors:** Phanupong Phutrakool, Krit Pongpirul

**Affiliations:** 1 School of Global Health and Department of Preventive and Social Medicine, Faculty of Medicine, Chulalongkorn University, 1873 Rama IV Road, Patumwan, Bangkok, 10330 Thailand; 2 Department of International Health and Department of Health, Behavior and Society, Johns Hopkins Bloomberg School of Public Health, Baltimore, MD USA; 3 Bumrungrad International Hospital, Bangkok, Thailand

**Keywords:** Acceptance, Use, Complementary and alternative medicine, Medical specialist

## Abstract

**Background:**

Complementary and Alternative Medicine (CAM) has gained popularity among the general population, but its acceptance and use among medical specialists have been inconclusive. This systematic review aimed to identify relevant studies and synthesize survey data on the acceptance and use of CAM among medical specialists.

**Methods:**

We conducted a systematic literature search in PubMed and Scopus databases for the acceptance and use of CAM among medical specialists. Each article was assessed by two screeners. Only survey studies relevant to the acceptance and use of CAM among medical specialists were reviewed. The pooled prevalence estimates were calculated using random-effects meta-analyses. This review followed both PRISMA and SWiM guidelines.

**Results:**

Of 5628 articles published between 2002 and 2017, 25 fulfilled the selection criteria. Ten medical specialties were included: Internal Medicine (11 studies), Pediatrics (6 studies), Obstetrics and Gynecology (6 studies), Anesthesiology (4 studies), Surgery (3 studies), Family Medicine (3 studies), Physical Medicine and Rehabilitation (3 studies), Psychiatry and Neurology (2 studies), Otolaryngology (1 study), and Neurological Surgery (1 study). The overall acceptance of CAM was 52% (95%CI, 42–62%). Family Medicine reported the highest acceptance, followed by Psychiatry and Neurology, Neurological Surgery, Obstetrics and Gynecology, Pediatrics, Anesthesiology, Physical Medicine and Rehabilitation, Internal Medicine, and Surgery. The overall use of CAM was 45% (95% CI, 37–54%). The highest use of CAM was by the Obstetrics and Gynecology, followed by Family Medicine, Psychiatry and Neurology, Pediatrics, Otolaryngology, Anesthesiology, Internal Medicine, Physical Medicine and Rehabilitation, and Surgery. Based on the studies, meta-regression showed no statistically significant difference across geographic regions, economic levels of the country, or sampling methods.

**Conclusion:**

Acceptance and use of CAM varied across medical specialists. CAM was accepted and used the most by Family Medicine but the least by Surgery. Findings from this systematic review could be useful for strategic harmonization of CAM and conventional medicine practice.

**Systematic review registration:**

PROSPERO CRD42019125628

**Graphical abstract:**

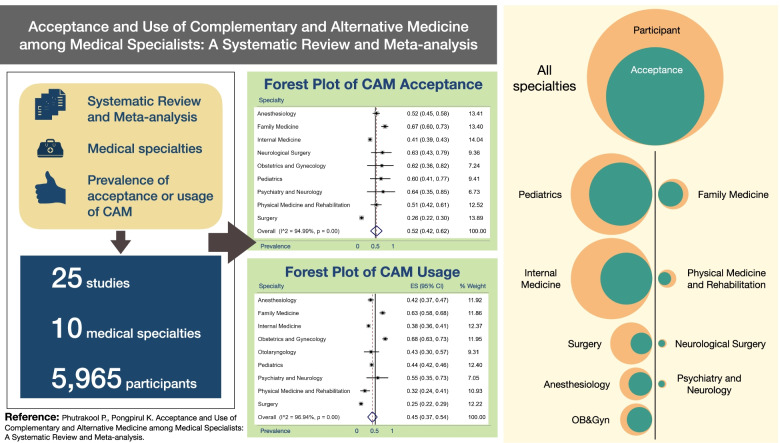

**Supplementary Information:**

The online version contains supplementary material available at 10.1186/s13643-021-01882-4.

## Background

Medical specialist is a healthcare professional who has undertaken specialized medical studies to diagnose, treat and prevent illness, disease, injury, and other physical and mental impairments in humans, using specialized testing, diagnostic, medical, surgical, physical, and psychiatric techniques, through application of the principles and procedures of modern medicine [1]. The specialized and general medical care have dominated as ‘conventional’ medical care in several countries, including Thailand.

Complementary and Alternative Medicine (CAM) is defined as medicine or treatment which is not considered as conventional (standard) medicine. The National Center for Complementary and Integrative Health (NCCIH) categorized most types of complementary medicines under two categories: (1) natural products and (2) mind-body practices [2]. Natural products include herbs, vitamins, minerals, and probiotics whereas mind-body practices include yoga, chiropractic, massage, acupuncture, yoga, meditation, and massage therapy. Types of CAM may vary across studies, but they overlap in most senses.

CAM is used by people throughout the world. A study showed that the prevalence estimate of CAM usage from 32 countries from all regions of the world to be 26.4%, ranging from 25.9 to 26.9%. For example, in 2013, the prevalence use of CAM in Australia, the USA, UK, and China were 34.7%, 21.0%, 23.6%, and 53.3%, respectively. The prevalence estimate of CAM satisfaction was as high as 71.9%, ranging from 71.0 to 72.7% [3].

Although patients are highly satisfied with CAM treatment, professional health care providers who are medical doctors do not offer CAM because it is not part of the standard conventional medical care. Although the majority of physicians who have used CAM were pleased with the results [4–8] and were more likely to recommend it to patients, friends, and family [9, 10] as a non-toxic treatment option; less than one third of the medical doctors were very comfortable in answering questions about CAM [9, 11–13] so patients who do not have the option to use CAM instead of standard medical care might be lost to follow-up. Some doctors are still skeptical of CAM because of a lack of specific knowledge and qualification as well as a lack of evidence from high-quality experimental studies on the efficacy of the CAM treatments [4, 12, 14, 15]. In the field of oncology, for example, the 5-year survival rate of breast cancer patients who refused standard treatment was 43.2%, compared with 81.9% of those who underwent the standard treatment [16]. When CAM was used, the 5-year survival rate was significantly worse. The 5-year survival rate of cancer patients who used CAM versus those who used standard treatment were stratified by cancer type were as follows: [17] for breast cancer 58.1% vs 86.6% (*p* value < 0.01; HR = 5.68), lung cancer 19.9% vs 41.3% (*p* value < 0.01; HR = 2.17), and colorectal cancer 32.7% vs 79.4% (*p* value < 0.01; HR = 4.57). On the contrary, the 28-day mortality of patient with sepsis and acute gastrointestinal injury who received CAM bundle with conventional therapy was statistically significantly lower than those who received only conventional therapy (21.2% vs 32.5%, *p* value = 0.038) [18]. These differential clinical benefits of CAM across various medical specialties could be partly explained by how CAM is perceived by the medical specialists in conventional medicine dominated contexts.

Several studies have surveyed the acceptance and use of CAM from laypersons [19–22] to healthcare professional perspectives [23–29]. Nonetheless, these surveys did not cover all medical specialists so the findings could not reflect the comparative acceptance and use of CAM across medical specialties. Also, previous studies could not determine whether the acceptance and use of CAM by medical specialists differ across contexts (i.e., regions and economic levels of the country) and study designs (i.e., survey and sampling methods). A better understanding of how various medical specialists perceive of CAM is strategically essential for harmonizing CAM into conventional medicine practices. This systematic review aimed to identify relevant studies and synthesize survey data on the acceptance and use of CAM among medical specialists.

## Materials and methods

### Protocol and registration

This systematic review has been registered in PROSPERO (CRD42019125628) and the protocol can be accessed at http://www.crd.york.ac.uk/PROSPERO/display_record.asp?ID=CRD42019125628.

### Literature search

This systematic review was conducted and reported according to the PRISMA statement as well as the Synthesis Without Meta-analysis (SWiM) guidelines [30]. A systematic literature search was performed by two independent authors (PP and KP) using PubMed and Scopus databases. The search was limited to observational studies of human subjects and the English language. The medical specialist’s perspective related to CAM studies were focused. The search strategy was based on various combinations of words and focused on two main concepts: acceptance and usage of CAM. The last search was conducted on March 1, 2019.

For the PubMed database, the following combinations were applied: ("Traditional Medicine"[All Fields] OR "Alternative Medicine"[All Fields] OR "Complementary Medicine"[All Fields] OR "Acupuncture Therapy"[All Fields] OR "Holistic Health"[All Fields] OR "Homeopathy"[All Fields] OR "Spiritual Therapies"[All Fields] OR "Faith Healing"[All Fields] OR "Yoga"[All Fields] OR "Witchcraft"[All Fields] OR "Shamanism"[All Fields] OR "Meditation"[All Fields] OR "Aromatherapy"[All Fields] OR "Medical Herbalism"[All Fields] OR "Mind-Body Therapies"[All Fields] OR "Laughter Therapy"[All Fields] OR "Hypnosis"[All Fields] OR "Tai Ji"[All Fields] OR "Tai Chi"[All Fields] OR "Relaxation Therapy"[All Fields] OR "Mental Healing"[All Fields] OR "Meditation"[All Fields]) AND ("Health care provider"[All Fields] OR "Health care providers"[All Fields] OR "Health personnel"[All Fields]) AND ("2002/01/01"[PDAT]: "2017/12/31"[PDAT]) AND "humans"[MeSH Terms].

For the Scopus database, the following combinations were applied: (ALL("Traditional Medicine") OR ALL("Alternative Medicine") OR ALL("Complementary Medicine") OR ALL("Acupuncture Therapy") OR ALL("Holistic Health") OR ALL("Homeopathy") OR ALL("Spiritual Therapies") OR ALL("Faith Healing") OR ALL("Yoga") OR ALL("Witchcraft") OR ALL("Shamanism") OR ALL("Meditation") OR ALL("Aromatherapy") OR ALL("Medical Herbalism") OR ALL("Mind-Body Therapies") OR ALL("Laughter Therapy") OR ALL("Hypnosis") OR ALL("Tai Ji") OR ALL("Tai Chi") OR ALL("Relaxation Therapy") OR ALL("Mental Healing") OR ALL("Meditation")) AND (ALL("Health care provider") OR ALL("Health care providers") OR ALL("Health personnel")) AND PUBYEAR AFT 2001 AND PUBYEAR BEF 2018 AND DOCTYPE(ar) AND INDEXTERMS("Humans")

### Selection of studies

The titles and abstracts of the primary studies identified in the electronic search were screened by the same two authors. Duplicated studies were excluded. For the meta-analysis, the following inclusion criteria were set: (1) medical specialist’s perspective, (2) prevalence of acceptance or usage of CAM, (3) observational study design, and (4) published between 2002 to 2017. The following exclusion criterion was set: (1) Not relevant to the practice. We contacted the authors for studies that had incomplete and unclear information. If the authors did not respond within 14 days, we proceeded to analyze the data we had. Any disagreement was resolved through discussion and the final determination was made by the first author (PP).

### Data extraction and management

Two authors worked independently to review and extract the following variables: (1) general information, including the name of the studies, authors, and publication year, (2) characteristics of the studies, including the design of the studies, sampling method, country, and setting, (3) characteristics of the participants, including sample size, response, and type of specialty, and (4) outcomes, including the prevalence of acceptance, and usage of CAM. All relevant text, tables, and figures were examined for data extraction. Discrepancies between the two reviewers were resolved by the first author (PP).

### Study quality/risk of bias

We used the tool developed by Hoy et al. [31] to evaluate the study quality/risk of bias of the studies included in the analysis. The tool has 11 items: (1) national representativeness, (2) target population representativeness, (3) random selection or census undertaken, (4) minimal non-response bias, (5) data collection direct from the subject, (6) definition of the case used, (7) valid and reliable instrument, (8) the same mode of data collection for all subjects, (9) length of shortest prevalence period, (10) appropriate numerator and denominator used, and (11) summary assessment. Items 1 to 4 assessed the external validity, items 5 to 10 assessed the internal validity, and items 11 evaluated the overall study quality/risk of bias. Each item was assigned a score of 1 (high quality/low risk) or 0 (low quality/high risk), and the scores were summed to generate an overall quality score that ranged from 0 to 10. According to the overall score, we classified the studies as having a high quality/low risk of bias (>6), moderate quality/risk of bias (4 to 6), and low quality/high risk of bias (<4). Two authors (PP and KP) independently assessed the study quality/risk of bias and any disagreement was resolved by discussion and consensus.

### Conflict of interest

We assessed the conflict of interest of the authors’ declarations in the studies.

### Statistical analysis

Unadjusted prevalence estimates of acceptance and usage of CAM were calculated based on the information of crude numerators and denominators provided by the studies and medical specialty [32]. Pooled prevalence was estimated from the prevalence as reported by the eligible studies. For each study and specialty, forest plots were generated displaying the prevalence with a 95% CI. The overall random-effects pooled estimate with its 95% CI were reported. To examine the magnitude of the variation between the studies, we quantified the heterogeneity by using *I*^*2*^ and its 95% CI.

To assess the level of heterogeneity as defined in Chapter 9 of the Cochrane Handbook for Systematic Reviews of Interventions, the following *I*^*2*^ cut-offs for 0 to 40% represented that the heterogeneity may not be important, 30 to 60% may represent moderate heterogeneity, 50 to 90% may represent substantial heterogeneity, and 75 to 100% represented that there was a considerable heterogeneity. For the *X*^*2*^ test, statistical heterogeneity of the included trials was assessed with a *p* value of less than 0.05 (statistically significant). The random-effects meta-analysis by DerSimonian and Laird method was used, and statistical heterogeneity was encountered. The meta-analysis was performed using Stata/MP software version 15 (StataCorp 2017, College Station, TX).

### Additional analysis

Meta-regression was performed to investigate the pooled prevalence differences between various regions (African region, region of the Americas, Eastern Mediterranean region, European region, Southeast Asia region, Western Pacific region, and mixed region) [33], economic levels of the country (low-income, lower-middle-income, upper-middle-income, high-income, and mixed-income) [34], and the sampling method (random and convenience sampling) for each study.

## Results

### Selection of the studies

**Fig. 1:**
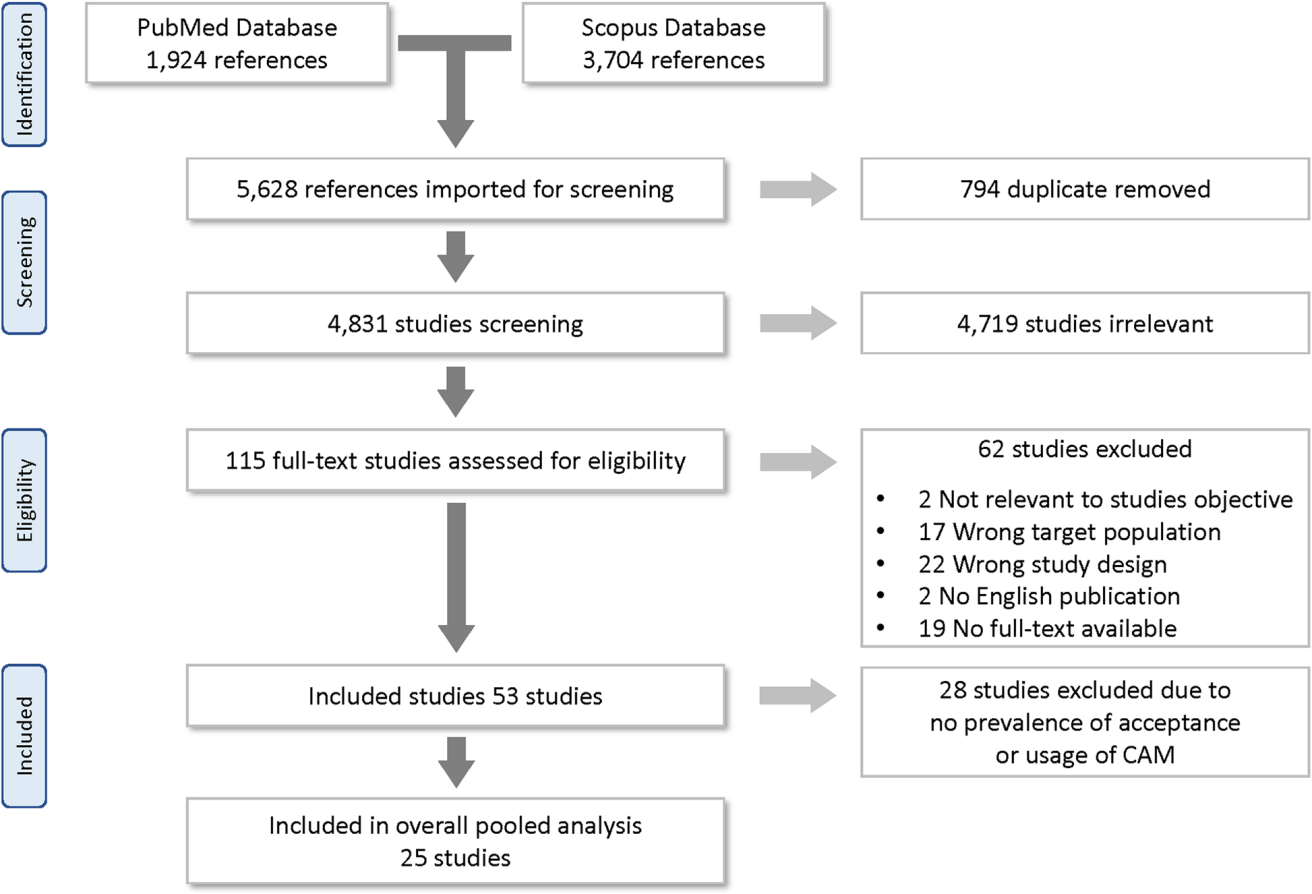
Selection of the studies

The literature search yielded 5628 articles. After 794 duplicates were removed, 4831 titles and abstracts were screened, and 4719 irrelevant articles were removed. Of 115 articles selected for full-text screening, 62 were excluded for the following reasons: two were not relevant to this study’s objective, 17 had the wrong target population, 22 did not have the study design required for this review, two study was not published in English, 19 did not have full-text available, and 28 did not provide the prevalence. Finally, a total of 25 articles, published between 2002 and 2017, fulfilled the selection criteria and were included in this meta-analysis (Fig. 1).

### Characteristics of the studies

All included studies were cross-sectional. The publication years ranged from 2002 to 2017 in various countries: European region (*n* = 11, 44%), region of the Americas (*n* = 10, 40%), Western Pacific region (*n* = 3, 12%), and mixed region (*n* = 1, 4%). Twenty-three studies (88%) were from high-income countries, 2 (8%) from upper-middle income countries, and 1 (4%) was from mixed-economic level country. The included studies indicated which type of collection method was used: online survey (*n* = 8, 32%), postal survey (*n* = 8, 32%), online and postal survey (*n* = 3, 12%), online and phone survey (*n* = 1, 4%), and the collection method was not reported (*n* = 5, 20%). The studies included a total of 7320 participants who were categorized as medical specialty (*n* = 5445, 74%), and non-medical specialty (*n* = 1875, 26%) (Table 1).

**Table 1:** Characteristics of the included studies

First author	Year	Country	Setting	Sampling method	Survey method	Response, %	Response, *n*	Medical specialist, *n*	Citation
Rosenbaum	2002	USA	The University of Iowa College of Medicine	Random	Postal	18.4%	690	265	[35]
Hyodo	2003	Japan	The Japanese oncology literature and the Nationwide association of medical centers for cancer and adult diseases	Random	Postal	66.7%	54	52	[36]
Kemper	2004	USA	The American Academy of Pediatrics	Random	Online	19.5%	195	195	[37]
Kolstad	2004	Norway	Five university oncology units in Norway	Random	Postal	38.5%	751	751	[5]
Risberg	2004	Norway	Five reginal oncology centers	Random	Postal	15.4%	104	104	[38]
Samano	2005	Brazil	Effective physician members of the Brazilian Cancer Society	Random	Postal	61.5%	509	108	[39]
Sawni	2007	USA	The American Academy of Pediatrics	Random	Postal	31.5%	268	263	[40]
Lee	2008	USA, China, and Taiwan	The Northern California Tumor Board meeting, China Medical University, Sun-Yat Sen Cancer Center Taiwan, Peking University Cancer Hospital China, and Peking Union Hospital China	Random	Postal	38.0%	95	95	[15]
Mak	2009	Australia	The Australasian Faculty of Rehabilitation Medicine, The Royal Australasia College of Physicians	Random	Online	38.3%	36	36	[41]
Wu	2009	USA	The Washington State of Association of Neurological Surgeons	Random	Online	67.0%	65	65	[42]
Manek	2010	USA	The practicing rheumatologists in the United States	Random	Postal	40.3%	381	381	[6]
Kundu	2011	USA	The Seattle Children’s Hospital	Random	Online	43.7%	213	213	[43]
Tempest	2011	England	A urologist practicing in three English training deaneries	Random	Online and Phone	13.4%	88	88	[44]
Vlieger	2011	Netherlands	The Dutch Society of Paediatrics	Random	Online	Not indicated denominator	170	170	[45]
Samuels	2013	Israel	Member of the Obstetricians and gynecologists (board-certified specialists or residents) were recruited from 7 medical centers in Southern, Central, and Northern Israel	Convenience	Not indicated	18.5%	648	648	[46]
Trimborn	2013	Germany	A German employee visiting the occupational health service of the university hospital	Convenience	Not indicated	75.7%	258	258	[8]
Conrad	2014	Germany	The German Society for Palliative Care	Random	Online	86.7%	117	40	[47]
Stewart	2014	Scotland	The care of pregnant women in the Grampian region of North-East Scotland	Random	Online and postal	72.0%	126	96	[48]
Brambila-Tapia	2016	Mexico	The Primary and secondary care hospitals in Guadalajara	Convenience	Not indicated	13.0%	547	120	[49]
Crundwell	2016	UK	The Clinical staff at Cambridge University Hospital’sotolaryngology and audiology departments	Convenience	Not indicated	23.7%	343	343	[10]
Gaboury	2016	Canada	The College des medecins du Quebec	Random	Online	100.0%	207	107	[50]
Mann	2016	USA	pain medicine fellowship at the American College of Graduate Medical Education	Convenience	Online and postal	53.3%	856	856	[7]
Soos	2016	Hungary	Four Hungarian universities and other eleven surgery wards and intensive care departments participated in the study	Convenience	Online and postal	61.5%	509	101	[51]
Stone	2016	Australia	All faculties, fellows, and residents presented at a single anesthesia grand rounds of Johns Hopkins University	Random	Not indicated	70.3%	102	102	[52]
Klein	2017	Germany	The Research Group on Gynecological Oncology of the German Cancer Society	Random	Online	38.1%	24	24	[14]

The included studies had the following medical specialties: internal medicine (11 studies, *n* = 2253), pediatrics (6 studies, *n* = 2,130), obstetrics and gynecology (6 studies, *n* = 707), anesthesiology (4 studies, *n* = 342), surgery (3 studies, *n* = 564), family medicine (3 studies, *n* = 296), physical medicine and rehabilitation (3 studies, *n* = 104), psychiatry and neurology (2 studies, *n* = 22), otolaryngology (1 study, *n* = 49), and neurological surgery (1 study, *n* = 24) (Table 2)

**Table 2:** The number of medical specialists according to the American Board of Medical Specialties

No.	American Board of Medical Specialties	Studies	Participants
1	Allergy and Immunology	-	-
2	Anesthesiology	4	342
3	Colon and Rectal Surgery	-	-
4	Dermatology	-	-
5	Emergency Medicine	-	-
6	Family Medicine	3	296
7	Internal Medicine	11	2,108
8	Medical Genetics and Genomics	-	-
9	Neurological Surgery	1	24
10	Nuclear Medicine	-	-
11	Obstetrics and Gynecology	5	326
12	Ophthalmology	-	-
13	Orthopaedic Surgery	-	-
14	Otolaryngology - Head and Neck Surgery	1	49
15	Pathology	-	-
16	Pediatrics	6	2,130
17	Physical Medicine and Rehabilitation	3	104
18	Plastic Surgery	-	-
19	Preventive Medicine	-	-
20	Psychiatry and Neurology	2	22
21	Radiology	-	-
22	Surgery	3	564
23	Thoracic Surgery	-	-
24	Urology	-	-
Total		39	5,965

### Based on the specialty

#### Prevalence of CAM acceptance

**Fig. 2:**
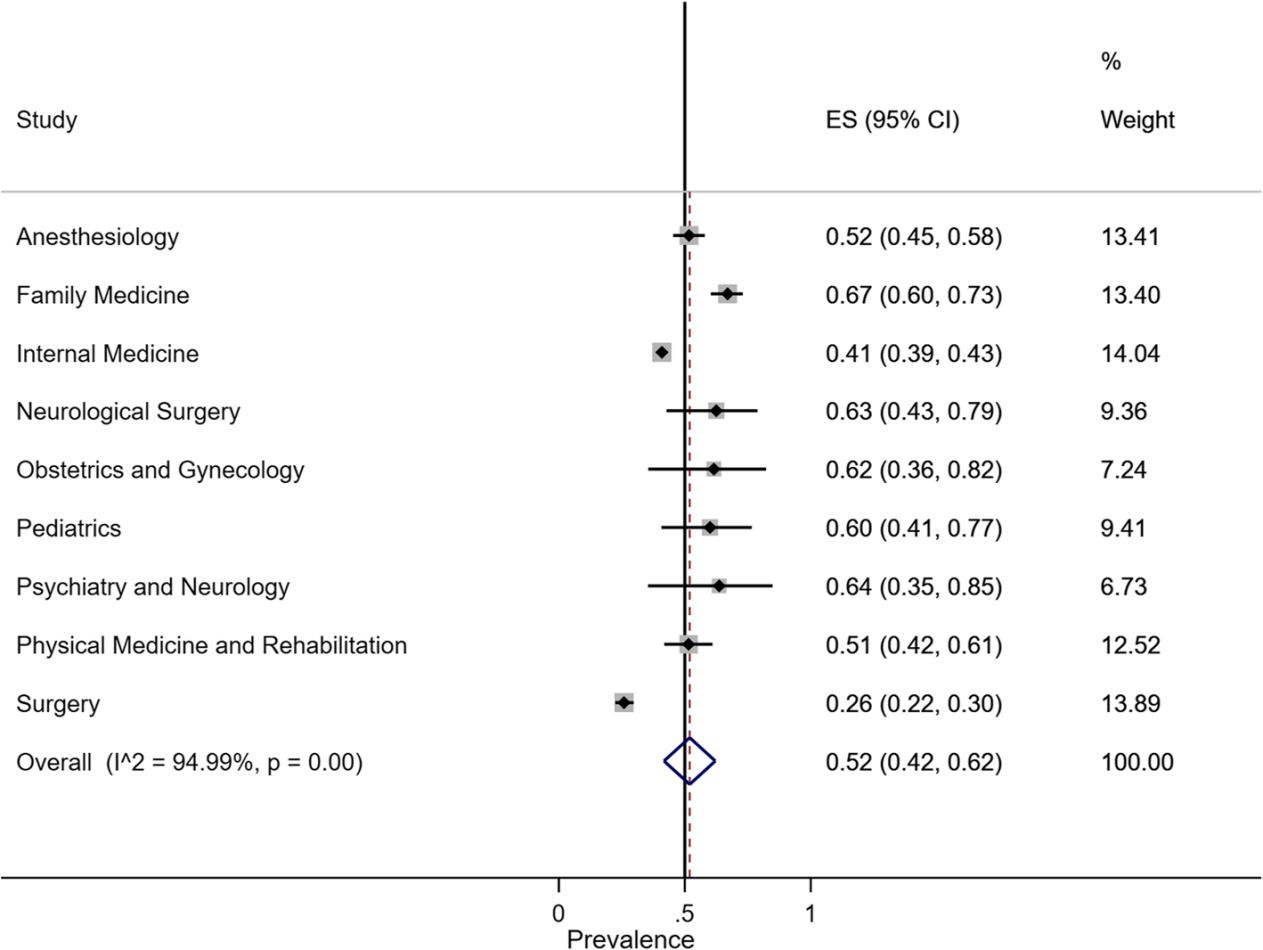
Forest plot of CAM acceptance by specialty

The overall random-effect pooled prevalence of CAM acceptance in medical specialty was 52% (95% CI, 42–62%). The prevalence of CAM acceptance in Family Medicine was 67% (95% CI, 60–73%), Psychiatry and Neurology was 64% (95% CI, 35–85%), Neurological Surgery was 63% (95% CI, 43–79%), Obstetrics and Gynecology was 62% (95% CI, 36–82%), Pediatrics was 60% (95% CI, 41–77%), Anesthesiology was 52% (95% CI, 45–58%), Physical Medicine and Rehabilitation was 51% (95% CI, 42–61%), Internal Medicine was 41% (95% CI, 39–43%), and Surgery was 26% (95% CI, 22–30%). The overall heterogeneity was significant (*I*^2^ = 94.99%, *p* value < 0.001) (Fig. 2).

#### Prevalence of CAM usage

**Fig. 3:**
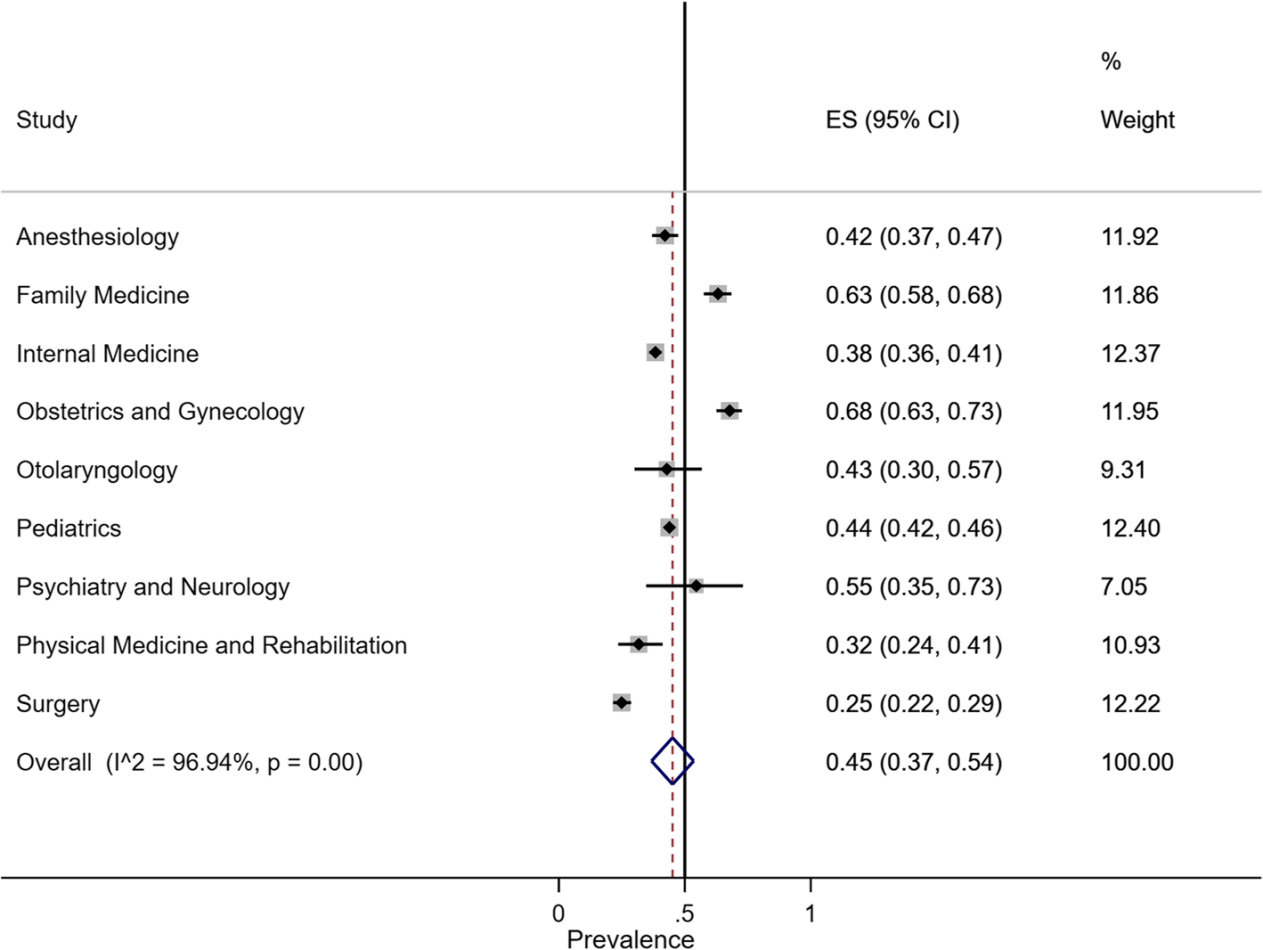
Forest plot of CAM usage by specialty

The overall random-effect pooled prevalence of CAM usage in medical specialty was 45% (95% CI, 37–54%). The prevalence of CAM usage in Obstetrics and Gynecology was 68% (95% CI, 63–73%), Family Medicine was 63% (95% CI, 58–68%), Psychiatry and Neurology was 55% (95% CI, 35–73%), Pediatrics was 44% (95% CI, 42–46%), Otolaryngology was 43% (95% CI, 30–57%), Anesthesiology was 42% (95% CI, 37–47%), Internal Medicine was 38% (95% CI, 36–41%), Physical Medicine and Rehabilitation was 32% (95% CI, 24–41%), and Surgery was 25% (95% CI, 22–29%). The overall heterogeneity was significant (*I*^2^ = 94.90%, *p* value < 0.001) (Fig. 3).

### Based on the studies

#### Prevalence of CAM acceptance

**Fig. 4:**
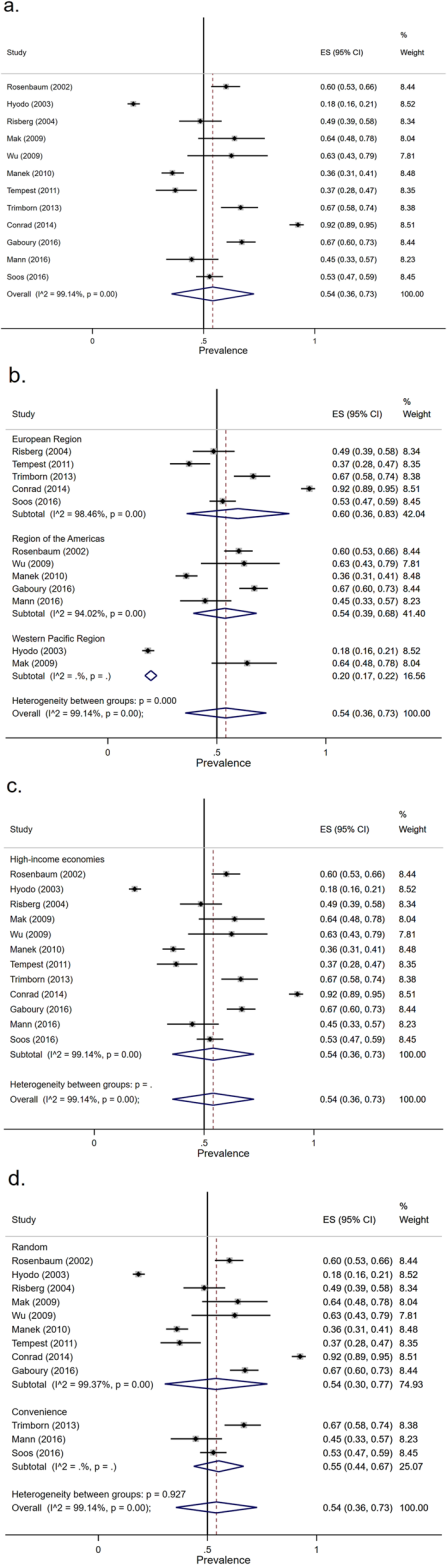
Forest plot of CAM acceptance

The overall random-effect pooled prevalence of CAM acceptance was 54% (95% CI, 36–73%) (Fig. 4a). Twelve studies provided CAM acceptance: five studies in the European region, five studies in the region of the Americas, and two studies in the Western Pacific region. The pooled prevalence of the European region, region of the Americas, and Western Pacific region that accepted CAM were 60% (95% CI, 36–83%), 54% (95% CI, 39–68%), and 20% (95% CI, 17–22%), respectively (Fig. 4b). All 12 studies were done in high-income economic countries (Fig. 4c). Based on the sampling method, the pooled prevalence of random sampling method, and non-random sampling method were 54% (95% CI, 30–77%), and 55% (95% CI, 44–67%), respectively (Fig. 4d). The overall heterogeneity was significant (*I*^2^ = 99.14%, *p* value < 0.001) as was the between-group heterogeneity (*p* value < 0.001). Meta-regression showed that there were no significant differences in the pooled prevalence of CAM acceptance by region, economic levels of the country, and the sampling method (Table 3).

**Table 3:** Subgroup analysis

Subgroup	No. of studies (no. of MS)	Difference in pooled prevalence (95% CI)	*p*-value
CAM acceptance			
Region			
Region of the Americas	5 (842)	0.00 (-0.28, 0.27)	0.979
European Region	5 (846)	0.10 (-0.16, 0.36)	0.405
Western Pacific Region	2 (787)	-0.18 (-0.51, 0.16)	0.271
Sampling method			
Random sampling	9 (2032)	-0.01 (-0.32, 0.30)	0.943
CAM usage			
Region			
Region of the Americas	8 (2435)	0.12 (-0.08, 0.31)	0.222
European Region	9 (1460)	0.04 (-0.16, 0.24)	0.706
Western Pacific Region	3 (883)	-0.17 (-0.45, 0.10)	0.204
Mixed region	1 (95)	-0.36 (-0.79, 0.07)	0.098
Economic levels of country			
Upper-middle-income economies	2 (195)	0.24 (-0.08, 0.56)	0.133
High-income economies	18 (4583)	-0.03 (-0.32, 0.25)	0.804
Mixed-income economies	1 (95)	-0.36 (-0.79, 0.07)	0.098
Sampling method			
Random sampling	15 (4101)	-0.03 (-0.25, 0.19)	0.802

*Abbreviations*: *CI* confidence interval

#### Prevalence of CAM usage

**Fig. 5:**
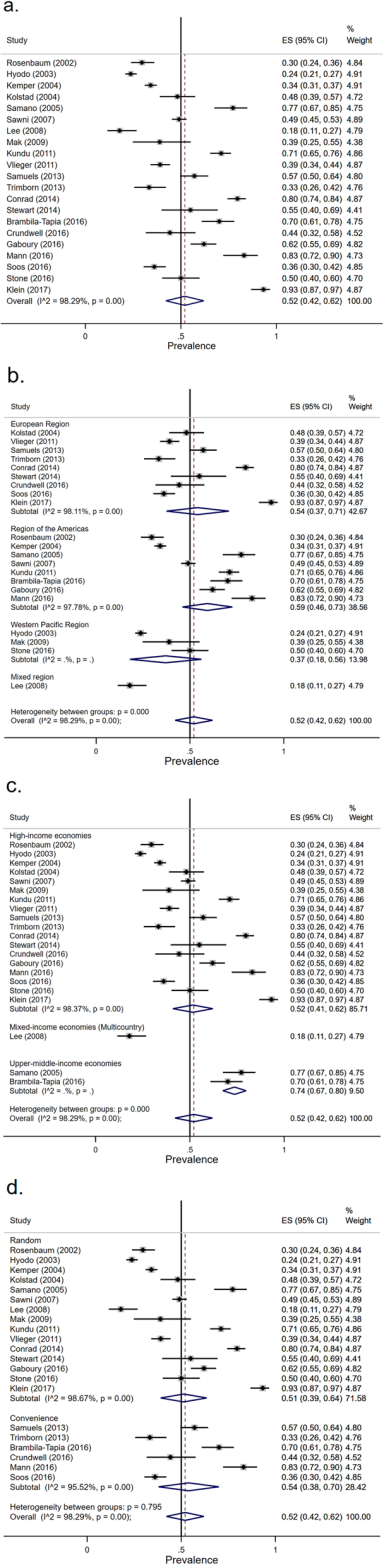
Forest plot of CAM usage

The overall random-effect pooled prevalence of CAM usage was 52% (95% CI, 42–62%) (Fig. 5a). Twenty-one studies provided CAM usage information: nine studies in the European region, eight studies in the region of the Americas, three studies in the Western Pacific region, and one study in the mixed region. The pooled prevalence of European region, region of the Americas, Western Pacific region, and mixed region that used CAM were 54% (95% CI, 37–71%), 59% (95% CI, 46–73%), 37% (95% CI, 18–56%), and 18% (95% CI, 11–27%), respectively (Fig 5b). All 18 studies were conducted in high-income economic countries, two studies were conducted in upper-middle-income economic countries, and one study was conducted in a mixed-income economic country. The pooled prevalence of high-income economic countries, upper-middle-income economic, and mixed-income economic countries that used CAM was 52% (95% CI, 41–62%), 74% (95% CI, 67–80%), and 18% (95% CI, 11–27%), respectively (Fig. 5c). Based on the sampling method, the pooled prevalence of the random sampling method, and non-random sampling method were 51% (95% CI, 39–64%), and 54% (95% CI, 38–70%), respectively (Fig. 5d). The overall heterogeneity was significant (*I*^2^ = 98.29%, *p* value < 0.001) as was between-group heterogeneity (*p* value < 0.001). Meta-regression showed that there were no significant differences in the pooled prevalence of CAM usage by region, economic levels of the country, and the sampling method (Table 3).

#### Assessment of study quality/risk of bias/conflict of interest

**Fig. 6:**
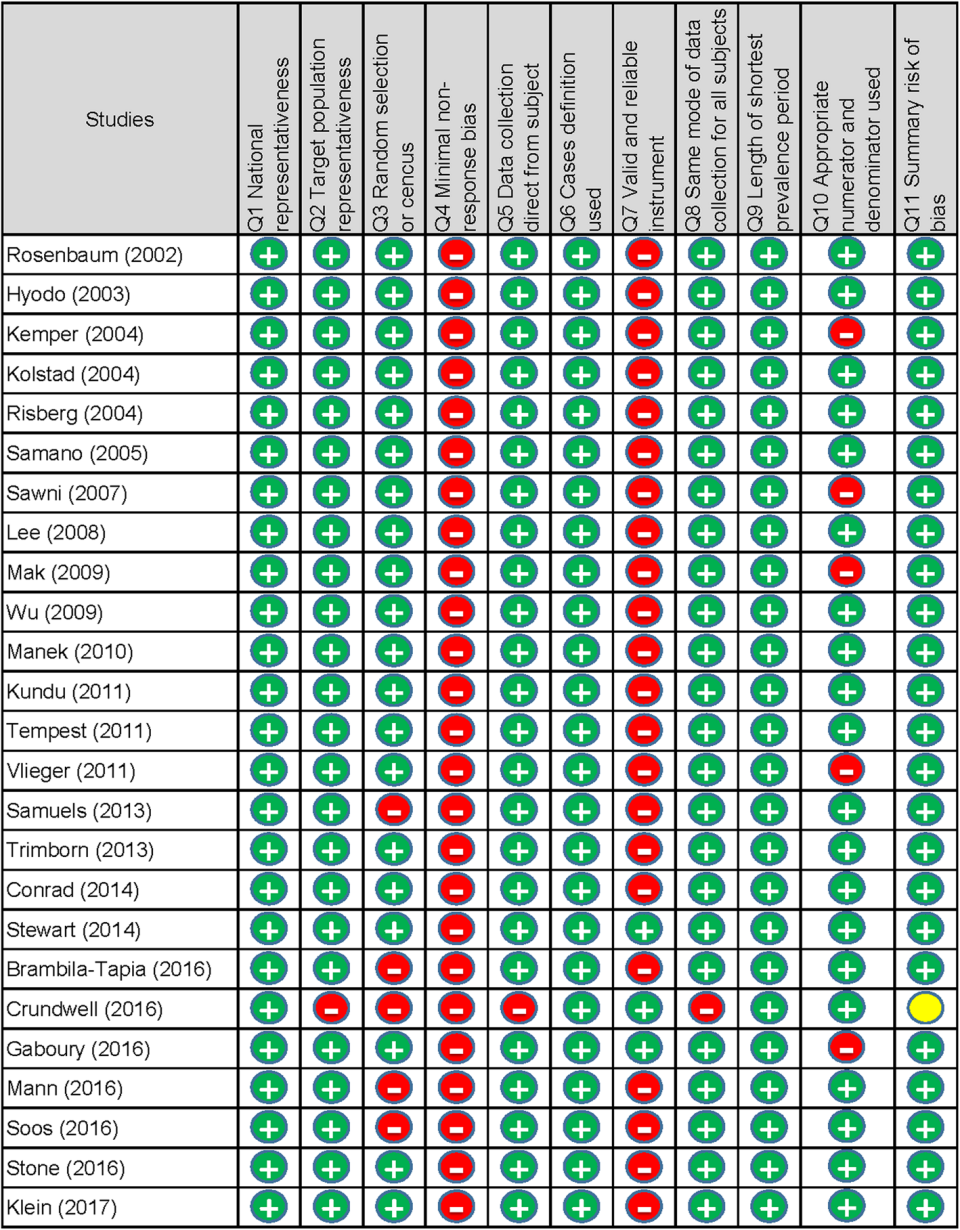
Study quality/risk of bias of the included studies

A total of 24 (96%) studies were categorized as high quality/low risk of bias, whereas one (4%) was categorized as moderate quality/moderate risk of bias. No study met the criteria of low quality/high risk of bias (Fig 6). Only five studies (20%) declared that there were conflicts of interest.

## Discussion

This study is the first of its kind to compare the acceptance and use of CAM across various medical specialties in different contexts. As nearly three-quarters of the specialties accepted CAM more than 50% whereas nearly a third were using CAM more than 50%.

The synthesis of all prevalence estimates of acceptance and usage was 52% and 45%, respectively. The highest prevalence of acceptance was in Family Medicine, followed by Psychiatry and Neurology, Neurological Surgery, Obstetrics and Gynecology, Pediatrics, Anesthesiology, Physical Medicine and Rehabilitation, Internal Medicine, and Surgery. The highest prevalence of usage was in Obstetrics and Gynecology, followed by Family Medicine, Psychiatry and Neurology, Pediatrics, Otolaryngology, Anesthesiology, Internal Medicine, Physical Medicine and Rehabilitation, and Surgery. These findings were useful in terms of improving care plan, decision-making processes, and communication in terms of CAM between the doctors and the patients.

All of the medical specialties mentioned above had a higher prevalence of acceptance than the prevalence of CAM use, except for Obstetrics and Gynecology because the gynecologic oncologists have used CAM to treat a large number of breast cancer patients [14]. There was a small difference in the prevalence (<5%) between the acceptance and the usage in Family Medicine (4%), Obstetrics and Gynecology (4%), Internal Medicine (3%), and Surgery (1%).

A highest difference of prevalence of CAM acceptance and usage was in the field of Physical Medicine and Rehabilitation (19%). This difference may be due to the reduction in the use of acupuncture in the academic hospitals [7] as well as personal use. Nearly two thirds of the rehabilitation physicians advised against the use of CAM as a therapeutic option [41]. The lowest prevalence of acceptance and usage of CAM was observed in Surgery. This relatively low prevalence compared to other medical specialties may be due to the belief that CAM products were ineffective. Many surgeons lacked information regarding CAM usage.

The acceptance of CAM was neutral in European region and region of the Americas. The World Health Organization reported that the prevalence of CAM usage in the European region, region of the Americas, and Western Pacific region in 2018 was 89%, 80%, and 95%, respectively [33], while this review found that the corresponding prevalence was 54%, 59%, and 37%, respectively. The lower prevalence may be from the dominating studies that were conducted before 2010 whereas CAM has used more often after 2010.

The variation of prevalence of CAM used was investigated in relation to the economic level of the countries. There was a higher prevalence of CAM use in the upper-middle-income economies than the high-income economies which may be due to cultural, historical influences, and implementation of CAM in the national health system as seen in Brazil [39] and Mexico [49].

Our study has some limitations that should be considered when interpreting the findings. Only two databases—PubMed and Scopus—were included so this review might have missed some relevant studies that were indexed elsewhere. Nonetheless, both databases were considered efficiently sufficient and most relevant to our research question within a specific domain [53]. While Web of Science and Scopus share several common features, Scopus is a relatively smaller database but covers more modern studies than Web of Science. The included studies did not cover some medical specialties that might have different acceptance and usage of CAM. Therefore, the prevalence of acceptance and usage of CAM in these populations need additional surveys. The prevalence of acceptance in some specialties like Neurological Surgery, Obstetrics and Gynecology, Otolaryngology, Pediatrics, and Psychiatry and Neurology was reported by a single study, thus limiting the generality of such findings. High heterogeneity of acceptance and usage of CAM between medical specialty referred to the variation in professional characteristic and practice, measurement methods, and study questionnaire. Most of the studies were from high-income economic countries. There were no studies from low-middle and low-income economic countries which is of concern. We found that no studies compared the relevant demographic characteristics between the responders and non-responders that would increase non-response bias when estimating the prevalence of CAM use. Although most of the studies demonstrated low risk of bias, over 88% of the studies did not use a validated instrument. Finally, the conflict of interest was not declared in more than 80% of the studies which may result in unintentional bias in the collection, analysis, and interpretation of the data. This can consequently lead to claims that the CAM used was beneficial because the researcher and/or entity may have a financial or management interest in the CAM used.

## Conclusions

Acceptance and use of CAM varied across medical specialties. Based on available survey data, CAM was accepted and used the most by Family Medicine but the least by Surgery. Findings from this systematic review could be useful for strategic harmonization of CAM and conventional medicine practice.

## Supplementary Information

**Additional file 1.** PRISMA Checklist.

**Additional file 2.** The citation for the Synthesis Without Meta-analysis explanation and elaboration article is: Campbell M, McKenzie JE, Sowden A, Katikireddi SV, Brennan SE, Ellis S, Hartmann-Boyce J, Ryan R, Shepperd S, Thomas J, Welch V, Thomson H. Synthesis without meta-analysis (SWiM) in systematic reviews: reporting guideline BMJ 2020;368:l6890 https://doi.org/10.1136/bmj.l6890.

## Data Availability

All data generated or analyzed during this study are included in this published article.
